# County-Level Hypertension Prevalence and Control in the United States: A ZIP3-County Crosswalk Using Electronic Health Record Data

**DOI:** 10.5888/pcd21.240185

**Published:** 2024-12-05

**Authors:** Xingran Weng, Adam S. Vaughan, Siran He, Angela M. Thompson-Paul, Rebecca C. Woodruff, Sandra L. Jackson

**Affiliations:** 1 Epidemic Intelligence Service, Centers for Disease Control and Prevention, Atlanta, Georgia; 2 Division for Heart Disease and Stroke Prevention, National Center for Chronic Disease Prevention and Health Promotion, Centers for Disease Control and Prevention, Atlanta, Georgia; 3 United States Public Health Service Commissioned Corps, Rockville, Maryland

**Figure:**
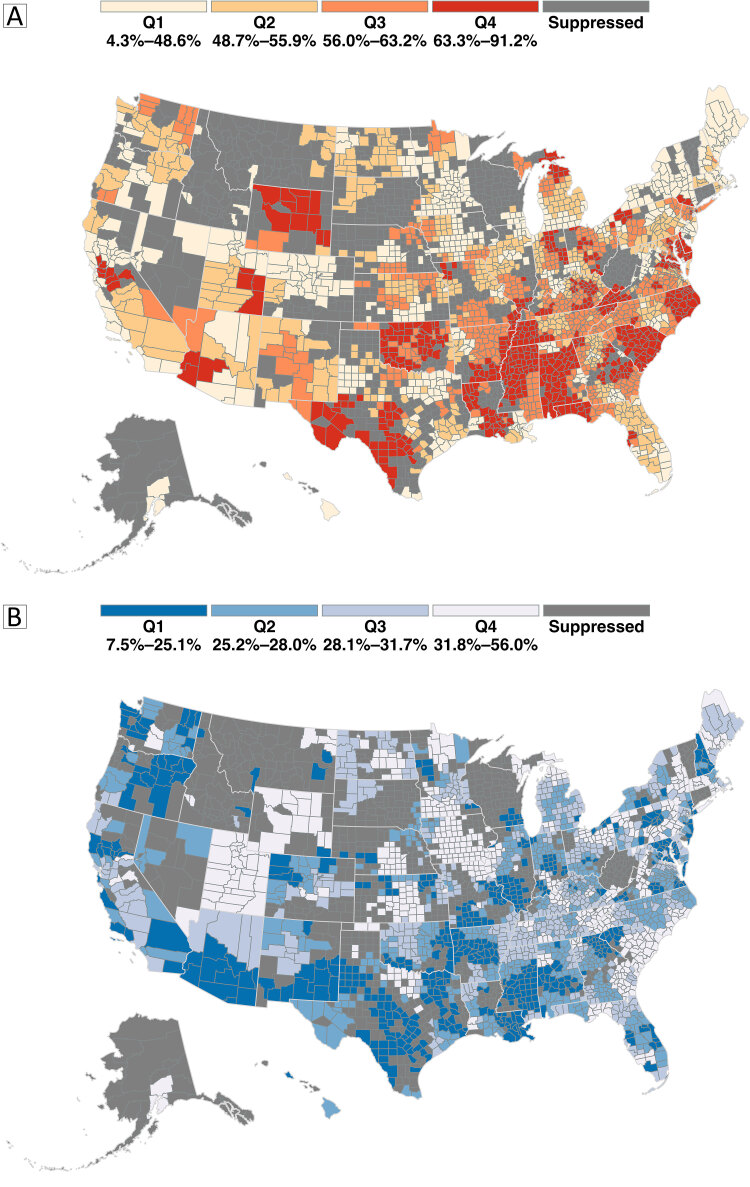
Prevalence of hypertension and hypertension control among US adult patients aged 18 years or older in 2022. Maps display county-level prevalence in quartiles of hypertension (Map A) and hypertension control (Map B) among adult patients in the IQVIA Ambulatory Electronic Medical Record-US database (1) in 2022. On Map A, the darker the color, the higher the hypertension prevalence, and on Map B, the darker the color, the lower the hypertension control prevalence among adults with hypertension. Suppressed counties (those with estimates that did not meet the National Center for Health Statistics rules) (2) were colored in dark gray on both maps.

## Purpose

Hypertension, which affects nearly half of US adults, is a modifiable risk factor for cardiovascular disease (3). Lifestyle and medication therapies can manage blood pressure, yet only a quarter of US adults with hypertension have the condition controlled (4). To guide hypertension management programs, local public health officials need county-level data. However, existing surveillance systems are not designed to produce hypertension prevalence and control estimates at such a granular geographic level (5,^6^). 

The National Health and Nutrition Examination Survey (NHANES) uses clinical data to produce national and regional estimates of hypertension prevalence and control, but not for individual counties. The Behavioral Risk Factor Surveillance System (BRFSS) can produce state-level estimates of self-reported hypertension awareness or county-level hypertension awareness derived from multilevel regression and poststratification disaggregating of state-level data (7). However, BRFSS does not collect data on hypertension control, and modeled estimates involve statistical assumptions that could potentially introduce additional bias into final county-level hypertension awareness estimates.

To complement existing surveillance systems, national electronic health record (EHR) outpatient data with detailed clinical information can be used to generate guideline-adherent and timely estimates of hypertension prevalence and control (3). Although EHR data may be available only at the 3-digit zip code (ZIP3) level, a methodology has been established to crosswalk ZIP3 to county-level estimates (8). We aimed to apply this crosswalk methodology and to explore the utility of EHR data for generating county-level estimates of hypertension prevalence and control.

## Data and Methods

To fully capture eligible patients, our study used patient-level EHR data from the IQVIA Ambulatory Electronic Medical Record-US (IQVIA AEMR-US, August 2023 release) database in the Observational Medical Outcomes Partnership Common Data Model format, from January 1, 2021, through December 31, 2022 (1). Annually, IQVIA AEMR-US captures roughly 11 million patients of all ages from over 100,000 providers across all 50 states and the District of Columbia. After implementing cohort inclusion criteria (9), a total of 7,581,012 adult patients aged 18 years or older in 2022 had valid ZIP3 data.

We used a published phenotype that uses EHR data to define hypertension by any of the following 3 criteria: 1) a diagnosis of hypertension, 2) two or more measurements of systolic blood pressure (BP) ≥130 mm Hg or diastolic BP ≥80 mm Hg from a patient’s vital signs (3), or 3) an antihypertensive medication prescription (9). Among people with hypertension, hypertension control status was identified as the most recent measurement of systolic BP <130 mm Hg and diastolic BP <80 mm Hg (3). All estimates were generated at the ZIP3 level, then ZIP3 estimates were crosswalked to the 2010 ZIP Code Tabulation Areas (ZCTA) level (assuming no discrepancy between ZCTA and ZIP5) (8). US Census Bureau data (including the proportion of ZCTA population in each county) were used to complete the ZIP3 to county crosswalk (8,10). County-level hypertension prevalence was then estimated by dividing the estimated number of adult patients with hypertension by the estimated number of total adult patients. County-level hypertension control was estimated by dividing the estimated number of adult patients with controlled hypertension by the total estimated number of adult patients with hypertension. All county-level estimates were classified into 4 quartiles (quartile 1, lowest hypertension prevalence or control, to quartile 4, highest hypertension prevalence or control). Counties with a population of less than 100 from the 2010 US Census and counties with estimates that did not meet the National Center for Health Statistics rules were suppressed (2).

We also calculated the Pearson correlation — comparing our county-level estimates of hypertension prevalence against county-level prevalence estimates of hypertension awareness from BRFSS (7) — for all counties and counties with a minimum population of 500 patients. Analyses were completed in R v4.2.2 (R Foundation for Statistical Computing).

## Highlights

Sufficient data were available to estimate hypertension prevalence and control in 2,264 of 3,143 US counties (72.0%). The median hypertension prevalence across all included counties was 55.9%. Counties in South Carolina, Alabama, Mississippi, Oklahoma, and Texas were in the highest hypertension prevalence quartile group (63.3%–91.2%).

Estimated median county-level hypertension control prevalence was 28.0%. Counties in the lowest hypertension control quartile (with prevalence ranging from 7.5% to 25.1%) were scattered mostly in the Southern, Midwestern, and Western states. Higher prevalences of hypertension control were observed in the Northeast and Mountain regions.

Taking both hypertension prevalence and control into consideration, areas with the highest hypertension prevalence and lowest hypertension control were the Mississippi Delta, southern Texas, western Oklahoma, southwestern Arizona, northeastern Georgia, and southern Illinois (Figure).

**Figure:**
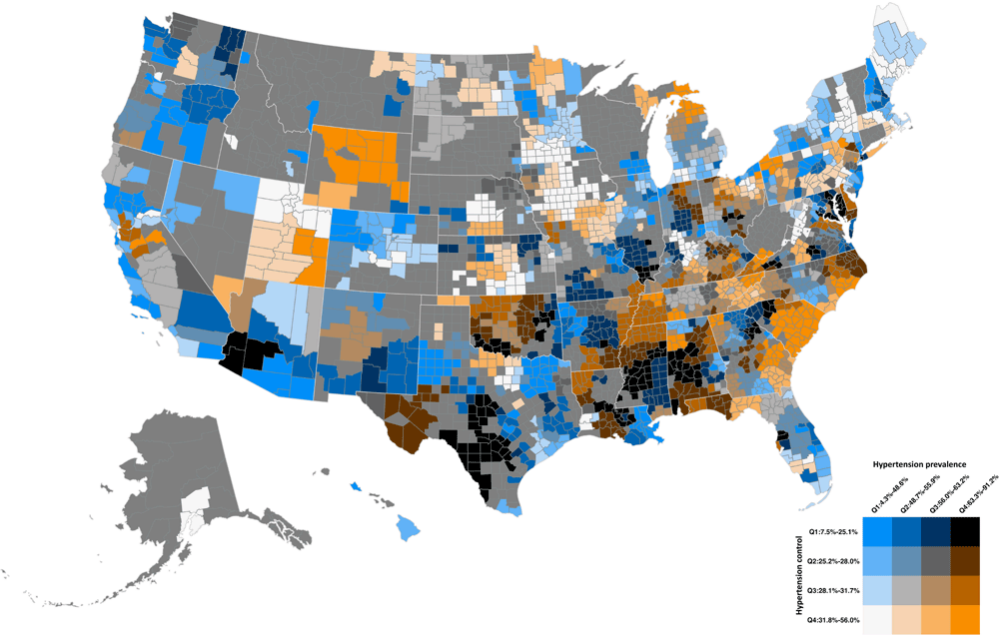
Bivariate map of hypertension prevalence and control among US adult patients aged 18 years or older in 2022.

Comparing our county-level hypertension estimates to BRFSS county-level hypertension awareness estimates (7), the correlation was 0.48. Restricting the sample to 1,032 counties with at least 500 estimated patients per county, the correlation increased to 0.55.

## Action

Our analysis of over 7 million patients in the IQVIA AEMR-US data system provides novel estimates of county-level hypertension prevalence and control derived from EHR data. Our granular and timely results provide policymakers, public health practitioners, and clinicians a first look at county-level hypertension control. By leveraging EHR data, this work may be used to tailor programmatic efforts; allocate resources; and inform hypertension prevention, screening, and control efforts among high-need communities. These efforts may then help to reduce hypertension burden, improve hypertension control, and subsequently improve cardiovascular health for US adults. Future hypertension surveillance could leverage these timely EHR data to track temporal trends and to continue identifying areas with a high disease burden.

Despite the potential utility of our results, county-level estimates should be interpreted with caution. EHR data reflect a care-seeking population, so hypertension prevalence and control estimates may be higher than estimates observed in a general population survey. Our results were based on assigning data from larger areas (ZIP3) to smaller areas (counties), which may have resulted in misclassification. Crosswalked results on maps may not fully reflect demographic patterns in the community. Additionally, existing county-level hypertension data were generated via estimation methodologies that may introduce biases, potentially explaining the modest correlation between our EHR-based estimates and PLACES county-level estimates (7).

In conclusion, results from this analysis suggest important geographic variability in hypertension prevalence and hypertension control among the care-seeking population, which can guide activities to reduce the nation’s hypertension burden.
